# Optimization of Mechanical Properties and Damage Tolerance in Polymer-Mineral Multilayer Composites

**DOI:** 10.3390/ma14040725

**Published:** 2021-02-04

**Authors:** Johannes Wiener, Hannes Kaineder, Otmar Kolednik, Florian Arbeiter

**Affiliations:** 1 Materials Science and Testing of Polymers, Montanuniversitaet Leoben, 8700 Leoben, Austria; johannes.wiener@unileoben.ac.at; 2 Institute of Polymer Extrusion and Compounding, Johannes Kepler University Linz, 4040 Linz, Austria; hannes.kaineder@jku.at; 3 Erich-Schmid-Institute of Materials Science, Austrian Academy of Science, 8700 Leoben, Austria; Otmar.Kolednik@oeaw.ac.at

**Keywords:** multilayer, biomimetic design, damage tolerance, polypropylene, microlayer

## Abstract

Talcum reinforced polypropylene was enhanced with a soft type of polypropylene in order to increase the impact strength and damage tolerance of the material. The soft phase was incorporated in the form of continuous interlayers, where the numbers of layers ranged from 64 to 2048. A blend with the same material composition (based on wt% of the used materials) and the pure matrix material were investigated for comparison. A plateau in impact strength was reached by layered architectures, where the matrix layer thickness was as small or smaller than the largest talcum particles. The most promising layered architecture, namely, 512 layers, was subsequently investigated more thoroughly using instrumented Charpy experiments and tensile testing. In these tests, normalised parameters for stiffness and strength were obtained in addition to the impact strength. The multilayered material showed remarkable impact strength, fracture energy and damage tolerance. However, stiffness and strength were reduced due to the addition of the soft phase. It could be shown that specimens under bending loads are very compliant due to a stress-decoupling effect between layers that specifically reduces bending stiffness. This drawback could be avoided under tensile loading, while the increase in toughness remained high.

## 1. Introduction

To use polymers in engineering applications successfully, a certain level of mechanical properties, such as stiffness, strength and impact strength, are required. Due to inherent limitations of the material properties, this is not always possible. Therefore, polymers are often reinforced with glass fibres, carbon fibres or mineral particles. Kausch et al., for example, found a strong increase in stiffness when adding alumina to polystyrene [1]. If surface treatment is performed correctly, mineral fillers can also increase the strength of a polymer matrix [2]. As a result of the reduced chain mobility, improvements were also found in the long-term creep behaviour [3,4]. Comparisons of the many useful combinations of matrices and reinforcements are available in extensive volumes for polymeric materials [5,6]. Unfortunately, high filler contents also lead to embrittlement, making these materials unusable for certain applications (e.g., when impact loading or high deformations are expected). Solutions must be found to counteract the embrittlement while preserving the benefits of the reinforcement. One approach is to maximize particle orientation parallel to the expected loading direction. While optimizing the load bearing capacity of the particles, this practice may also reduce defect size [7,8]. Another approach is to add a soft component as a toughening agent to increase the impact strength [9,10,11]. The conventional method for this is blending, e.g., by compounding, which leads to a random distribution of the soft phase. The change in properties roughly adheres to the rule of mixture, so that improvements in impact strength may come at the cost of stiffness and strength [9,10,11].

Alternatively, the soft phase can be incorporated as distinct domains, for example, as continuous layers in a co-extrusion process [12,13]. Studies on natural materials suggest great potential for alternating layers of stiff and compliant material [14,15,16,17]. Publications on nacre show its remarkable toughness, although its main component is brittle aragonite [18,19]. The skeleton of deep sea sponges also reveals astounding toughness and flexibility, especially considering that it is mostly made out of glass [20,21,22,23,24]. Bone is known to have outstanding strength-to-weight and stiffness-to-weight ratios [25] while also serving an organ-like function for the production of blood cells [26]. In most cases, a combination of high stiffness and high toughness in these materials can be traced back to intricate microstructures [27,28,29,30,31]. Small domains of protein-based soft phases within a stiff and brittle matrix phase (CaCO_3_, SiO_2_ etc.) act as toughness enhancers in these materials. Kolednik et al. showed that the soft domains are crucial for the increase in fracture toughness, as they encourage crack arresting mechanisms [32]. In nacre-like materials, crack deflection and platelet pull-out are able to further increase fracture toughness [33,34,35]. Replicating such microcomposites with commercial materials holds great potential for science and industry and is, therefore, worth investigating. One possibility would be the so-called material inhomogeneity effect, which can help to stop cracks at soft interlayers (IL). Various sources point out that a large difference in yield stress is required in order to diminish the crack driving force [32,36,37]. Such crack arresting properties of a soft IL have already been found in polymeric materials with one or two soft ILs [38,39,40].

To further deepen the understanding of this effect, and exhaust the possibilities of this mechanism, the properties of polypropylene/talcum composites with an increased number of layers (up to 2048) are investigated in this work. Several studies on micro- and nanolayer coextrusion already show the high potential of this method in multiple fields of research. Literature ranges from improved barrier properties [41,42,43] and flame retardancy [44] to altered crystallisation behaviour [45,46,47,48] and even semi-conductivity in metal filled polymers [49,50]. Publications on mechanical behaviour [7,8] report an increase in fracture strain, when average layer thickness is decreased below a certain critical threshold. To date, these studies have mainly focused on microstructural aspects of the multilayer materials, without further analysis of the trade-off between stiffness and toughness, which often has to be made [51,52]. In this contribution, we aim to offer a thorough investigation of the mechanical properties of a brittle matrix material, which are enhanced with different toughening techniques. A comparison between conventional blending, high particle orientation and a defined microstructure in the form of alternating layers is made regarding bending stiffness, impact strength and damage tolerance.

## 2. Materials and Methods

### 2.1. Specimen Preparation

**Figure 1:**
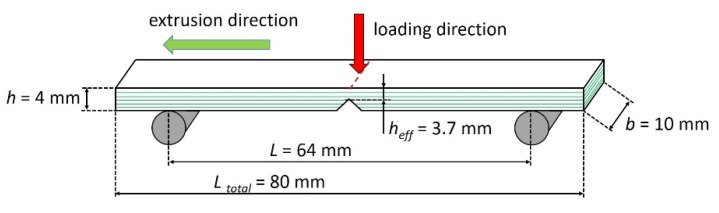
Charpy specimen dimensions with the notch introduced flatwise.

An overview of all investigated materials is summarized in Table 1. Therein, the material composition, a brief description as well as a schematic drawing of the microstructure are given. To accomplish the various layer architectures, a microlayer co-extrusion technique was used [12,13,53]. All materials were supplied in the form of extruded sheets with a thickness of 4 mm. From these, Charpy specimens according to DIN EN ISO 179-1 [54] were manufactured with different notch types (unnotched, Charpy-notched and razor blade notched according to [55,56,57]). As shown in Figure 1, all notches were introduced flatwise, so the effect of a layered plate could be observed. Additionally, tensile test specimens (Type 1A) were prepared in order to be tested according to DIN EN ISO 527 [58]. Detailed descriptions of the investigated materials as well as the processing technique are given in Appendix A.

### 2.2. Testing Methods

Uninstrumented as well as instrumented Charpy experiments were performed according to DIN EN ISO 179-1fA for notched and DIN EN ISO 179-1fU for unnotched specimens. An effective cross-sectional area, *A_eff_*, and the impact strength, *a_c_*, were calculated according to Equations (1) and (2). Therein, *b* is the specimen width, *h_eff_* the effective thickness and *U* the area under the force–displacement curve. Due to high oscillation during the instrumented impact tests, the maximum force, *F_max_*, and initial slope could not be reliably determined from the raw data. Therefore, a fit of cubic splines was created for each curve, which was then used to determine the maximum force and initial slope. The slope was calculated from the differences in force, Δ*F*, and displacement, Δ*v*, in the initial, linear part of the curve. From the initial slope in the unnotched state, the bending modulus, *E_b_*, was calculated according to Equation (3) to compare the stiffness of the materials. The calculation is in agreement with DIN EN ISO 178 [59], although the testing speed in this case is much higher than in conventional three-point-bending tests. *E_b_* should, therefore, be seen as a ranking parameter in these specific considerations and cannot be quantitatively compared to values obtained from quasi-static experiments. In order to remove the influence of specimen geometry, *F_max_* was divided by *A_eff_* of each individual specimen. The formula for the normalised maximum Force, $F_{max}^{norm}$, is shown in Equation (4).

(1) $$A_{eff}=\;bh_{eff}$$

(2) $$a_{c}=\;\frac{U}{A_{eff}}$$

(3) $$E_{b}\;=\;\frac{\Delta F}{\Delta v}\frac{L^{3}}{4bh\begin{array}{c} 3\\ eff \end{array}}$$

(4) $$F\begin{array}{c} norm\\ max \end{array}\;=\;\frac{F_{max}}{A_{eff}}$$

All tensile tests were performed according to DIN EN ISO 527, where the Young’s modulus, *E*, the ultimate tensile strength, *σ_UTS_*, and the elongation at break, *ε_br_*, were measured. In these tensile tests, the area under the force–displacement curve was corrected for elastic unloading and taken as fracture energy, which acted as a measure for toughness. To investigate the microstructure of the samples, scanning electron microscopy was applied, whereas the fractographs were generated from the backscatter electron to give an improved contrast between talcum particles and the polymer matrix. A more detailed description of all testing procedures is presented in Appendix A.

## 3. Results and Discussion

### 3.1. Impact Strength of Multilayer Composites

**Figure 2:**
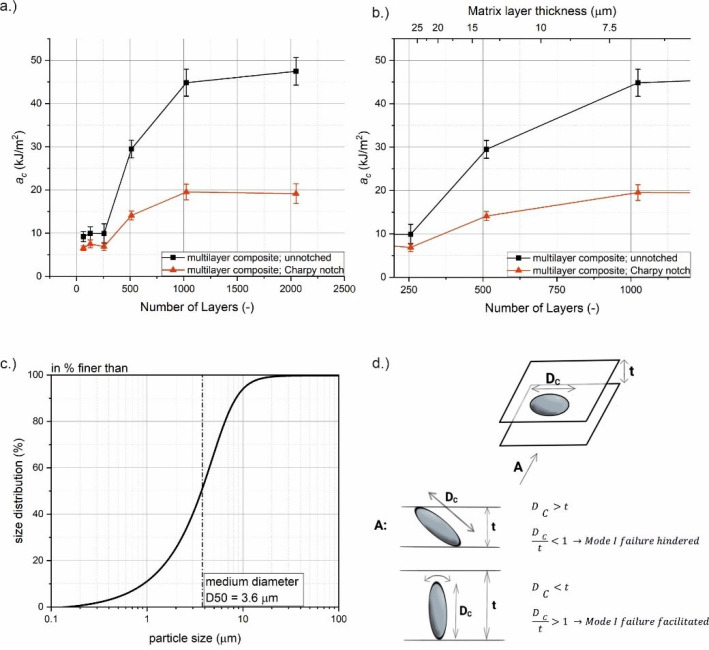
(**a**) Impact strength versus number of layers for a multilayer composite, (**b**) a magnification of the plateau region, (**c**) the size distribution of the talcum particles in the matrix and (**d**) a representation of large talcum particle orientation in a thin matrix layer.

In Figure 2a, the impact strength values of the layered composites are shown for Charpy notched and unnotched specimens. The numbers of layers range from 64 to 2048. Up to 256 layers, it appears that increasing the number of layers has no significant influence on the impact strength. However, increasing the number of layers beyond this point leads to a drastic increase in impact strength for both unnotched and Charpy notched specimens. At 1024 layers, the impact strength reaches a plateau of 45 kJ/m^2^ for unnotched and 20 kJ/m^2^ in the notched state. A further increase in the number of layers yields no improvements of impact strength. In comparison to the lower numbers of layers, this plateau value represents an increase in impact strength of approximately 4.5 times in the unnotched and three times in the notched state.

In Figure 2b, the transition region from the lower to the upper plateau is shown with greater magnification. The added scale at the top of Figure 2b shows the matrix layer thickness *t*. An explanation of this effect could be that the increased orientation of large talcum particles during the microlayering process improves the impact strength. No further improvement appears after all particles are oriented in extrusion direction. This improvement in impact strength might be also affected by the ratio of particle size to matrix layer thickness. The size distribution of talcum particles in the matrix is shown in Figure 2c. It is seen that the impact strength only increases when the matrix layer thickness falls below a critical dimension, *t < D_C_*. This critical dimension is the size of the largest talcum particles in the distribution, which are critical for failure when left unoriented. Thus, improvements of impact strength can be seen for matrix layer thicknesses of *t* < 13.6 μm. At this point, the matrix layers become smaller than the largest talcum particles which, therefore, have to be forcefully oriented during processing. As a result, these large particles are oriented along the layer plane, and their ability to act as critical defect for layer failure is diminished (Figure 2d). Note that the critical normal stress components for Mode I fracture lie in a plane perpendicular to the layer plane (Figure 1). If the matrix layer thickness is greater than *D_C_*, some of the large particles can be aligned perpendicular to the layer plane. If such a particle debonds from the matrix during impact loading, a large void oriented along the nominal fracture plane is formed. In that case, Mode I failure is facilitated, representing the worst-case scenario. Reducing the layer thickness below a certain threshold (approximately 7.8 μm in this specific case) brings no further improvement, since all large particles are already oriented. As a result, lower and upper plateaus in impact strength are formed.

Baer et al. [7] and Mueller et al. [8] found similar trends for talcum-filled PP composites, where the fracture strain started to increase drastically with an increasing number of layers. In these contributions, the altered material behaviour could also be linked to an increased degree of particle orientation. However, no lower plateau region was reported in these publications. Additionally, the fracture strain started to decrease again after a peak region instead of forming an upper plateau. The decrease in fracture strain at higher numbers of layers was attributed to particle agglomeration during processing. Mueller et al. even report quasi brittle fracture at 1024 layers or more. Up to 2048 layers, no such limitations could be found in the present investigation, indicating a high quality of processing. However, these sources agree that the limitation of defect size is responsible for the increased ductility along with a tortuous crack path, crack tip blunting and enhanced particle alignment [7]. Building on the results of this preliminary study, the most promising multilayer composite was selected for a more detailed investigation. More specifically, the composition with 512 layers (start of plateau region) was chosen for closer examination in further experiments.

### 3.2. Comparison of Single and 512-Layer Coextruded Materials

**Figure 3:**
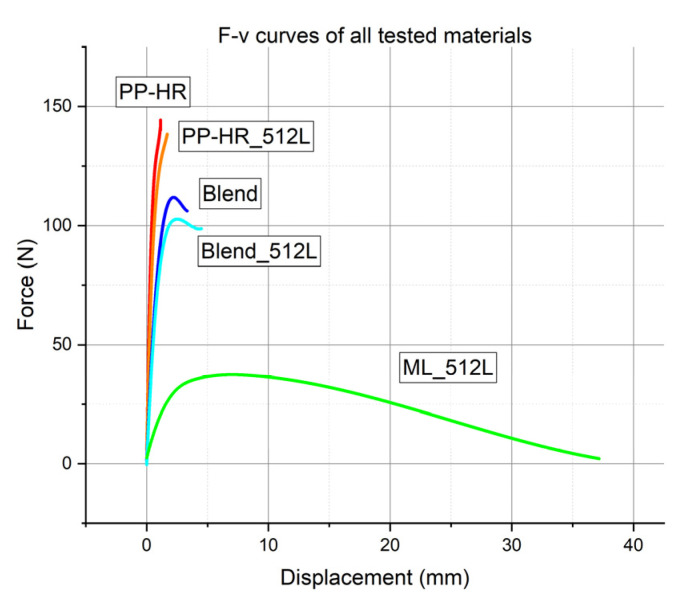
Representative force–displacement curves in instrumented Charpy experiments for unnotched materials.

**Figure 4:**
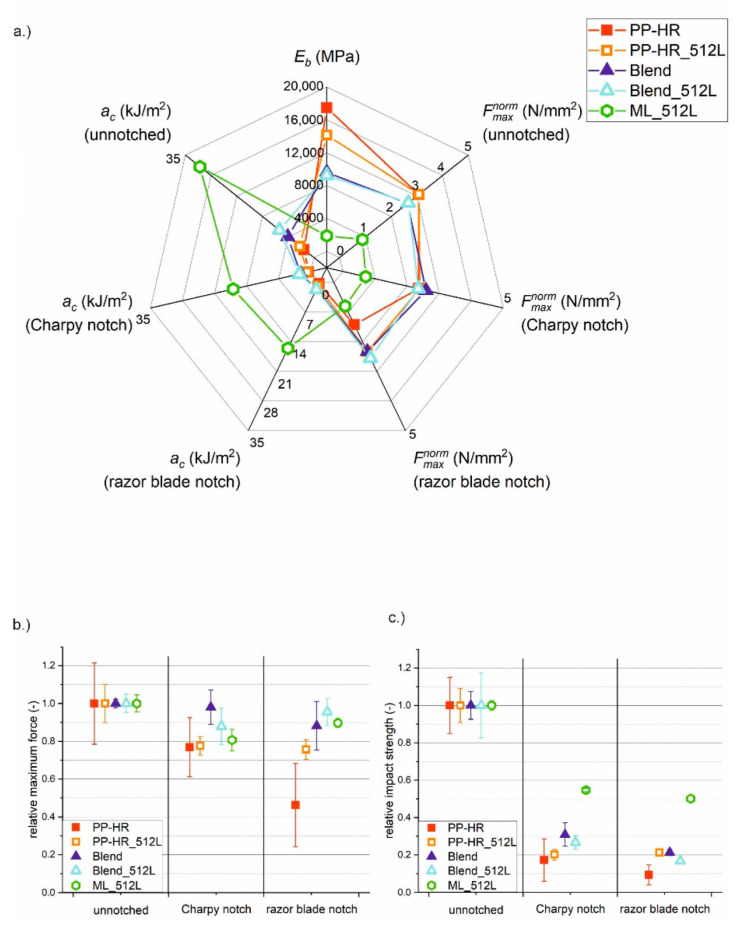
(**a**) Overview of *E_b_*, $F_{max}^{norm}$ and *a_c_* for unnotched, Charpy-notched and razor blade notched specimens of all tested materials. The notch sensitivity and data scattering of (**b**) $F_{max}^{norm}$ and (**c**) *a_c_* is depicted by referencing each value to the unnotched state of that material.

The transition region between the lower and upper plateau of impact strength is of special interest for understanding the changes in microstructure and failure mechanism. Hence, the multilayer material with 512 layers, ML_512L, was chosen for closer investigation instead of the composite with 1024 layers. Although ML_512L showed great results in the uninstrumented Charpy experiments, not much more is known of its material properties. It is not entirely clear yet whether the improvements in impact strength stem from particle orientation, the addition of soft phase or from the layered microstructure. Thus, ML_512L as well as comparable blends and the matrix material (see Table 1 for more details) were investigated more thoroughly in instrumented Charpy experiments. The experiments aim to provide a more detailed impression of material behaviour, including stiffness and damage tolerance. Representative force–displacement curves from the instrumented Charpy experiments are shown in Figure 3. The figure shows the fitted data from the unnotched specimens. *E_b_*, $F_{max}^{norm}$ and *a_c_* were obtained from the instrumented Charpy tests for all investigated materials and notch types. The results are given in full detail in Table A1 in Appendix B. A graphic representation of all obtained material parameters for unnotched, Charpy-notched and razor blade notched specimens is given in Figure 4a.

The force–displacement curves for PP-HR and PP-HR_512L (see Figure 3) resulted in high values of $F_{max}^{norm}$ and *E_b_* due to the high mineral content. However, high displacements could not be reached before the materials failed. For both homogeneous materials, the failure behaviour was a complete and brittle fracture without visible plastic deformation. PP-HR exhibited very low impact strength, especially in comparison to materials with a soft phase. The microlayering process of PP-HR_512L led to a minor improvement in *a_c_* over PP-HR, owing to a slight increase in elongation at break. However, the impact strength was still low compared to the blends and multilayer materials. These minor effects may be attributed to an increased degree of orientation, accompanied by a reduction in defect size. Additionally, *E_b_* of PP-HR_512L was reduced to 81% of PP-HR as a result of the microlayering process.

In order to give an impression of data scattering and notch sensitivity, the relative values for $F_{max}^{norm}$ and *a_c_* are plotted against the notch type in Figure 4b,c. The values for Charpy and razor blade notches are viewed in relation to the unnotched state of the same material, so the unnotched reference state was always given a value of 1. PP-HR was especially susceptible to any type of notching. The maximum force and impact strength were greatly decreased with increasing notch sharpness. The impact strength was even reduced by up to 90% in the presence of a razor blade notch. It is clear that such a material cannot be used for structural applications, since even small defects or scratches could lead to catastrophic failure under impact loading. PP-HR also showed the highest data scattering, which was probably caused by the size distribution of talcum particles. In the absence of a microlayering process, larger agglomerates of particles were not broken up during processing, leading to larger initial defects in the material. This may also have led to the low values of $F_{max}^{norm}$ and *a_c_* in the notched states. In the unnotched and Charpy notched state, PP-HR_512L showed roughly the same relative values of $F_{max}^{norm}$ and *a_c_*, but offered an improvement in the razor blade notched state. While PP-HR dropped to relative values of 50%, PP-HR_512L retained approximately 75% of $F_{max}^{norm}$. Regarding impact strength, PP-HR_512L retained 20% of *a_c_* in the presence of a sharp notch. The layering process also reduced the data scattering for PP-HR_512L, suggesting that very large agglomerates were broken up during the microlayering process. Thus, the variance in initial defect size was reduced, which led to more uniform values and less notch sensitivity.

The two blends had an overall reduced content of mineral filler by 13% compared to the matrix material. Therefore, $F_{max}^{norm}$ and *E_b_* were smaller than for PP-HR, but the impact strength of the matrix material could be drastically improved. While *E_b_* was reduced by approximately one third, the impact strength could be increased by 170% (Blend) and 250% (Blend_512L), respectively. As could already be assumed from the force–displacement curves, the microlayering process was beneficial to the ductility of the blend. One possible explanation for the increased ductility of Blend_512L is a more finely dispersed microstructure due to the microlayering process accompanied by an overall reduction in defect size. While the distribution of the matrix and soft phase was still random, large bulks of matrix-rich material were less likely. In the Blend, these areas were able to form a percolating network, which acted as a stiff but brittle skeleton within the material. As a result, the Blend_512L had increased impact strength for all three notch types, owing to its increased elongation at break. Although $F_{max}^{norm}$ appeared to be lower for Blend_512L at first glance (see Figure 3), this difference lies within the standard deviation of the measurement. However, both blends suffered from reduced impact strength when introducing a notch. As a result, impact strength was reduced by 75% for Charpy notches and 80% for razor blade notches. For both blends, the failure behaviour was a complete fracture with small plastic zones around the fracture plain. Although the soft phase increased the absolute level of impact strength, the material was almost as damage intolerant as the matrix material. Despite the domains of soft phase, the random microstructure was ill-suited to stop cracks from advancing.

For ML_512L, the failure behaviour was a partial fracture with large plastic zones around the crack tip. Due to the high compliance of ML_512L, the specimens were pulled through the Charpy fixture after a large amount of displacement, instead of breaking. Figure 3 shows the lowest forces, the highest displacements and a gradual reduction in force to 0 N as the specimens were pulled through the fixture. Despite the low values for $F_{max}^{norm}$ and *E_b_*, specimens with continuous interlayers had outstanding impact strength owing to the high displacements that could be endured. Although *E_b_* was reduced by a factor of 9.2 compared to PP-HR, the multilayer material had an impact strength over 60 times higher than the matrix material in the presence of a sharp notch (15.53 compared to 0.25 kJ/m^2^). Additionally, this multilayer composite showed the highest damage tolerance out of all investigated materials, retaining relative values of 80–90% for $F_{max}^{norm}$ for all notch types and 50% of *a_c_* in the razor blade notched state. These properties were not only caused by the addition of the soft phase, but also by the layered microstructure. Despite having the same material composition, ML_512L with a razor blade notch showed an impact strength twice as high as the unnotched Blend. Blends, on the other hand, exhibited comparably high stiffness, but could not offer the crack arresting properties associated with a multilayer microstructure. It is crucial that the soft phase is arranged in a defined structure stretching the whole cross section, e.g., as layers, so crack growth cannot avoid the soft domains. Thus, the multitude of soft layers could enhance damage tolerance through crack tip blunting and by limiting defect size.

### 3.3. Synergistic Effect of Layered Architecture

**Figure 5:**
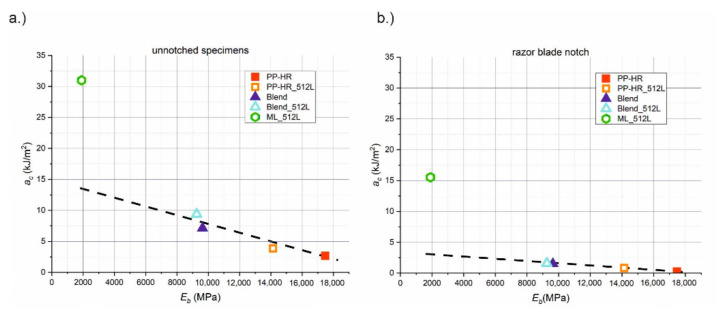
Comparison of stiffness and toughness showing impact strength versus the bending modulus *E_b_* for (**a**) unnotched and (**b**) razor blade notched specimens.

For a reasonable evaluation of a material, both toughness and stiffness need to be considered simultaneously. For that reason, the measured values for *a_c_* are plotted against *E_b_* in Figure 5. All unnotched materials are depicted in Figure 5a, while the razor blade notched materials are shown in Figure 5b. All observed trends remain qualitatively the same for notched and unnotched specimens. However, the absolute values of *a_c_* in the razor blade notched state are smaller. The values for Charpy notched specimens are not shown separately, since the difference in trends to the razor blade notched specimens is negligible. Similar to an Ashby plot, this depiction allows for a more comprehensive assessment of materials. PP-HR and PP-HR_512L are the stiffest, but also the most brittle materials. Apparently, the microlayering process increased the ductility of PP-HR_512L, thus increasing the impact strength while simultaneously decreasing *E_b_*. The two blends are not as stiff, but tougher than the homogeneous materials. The microlayered Blend_512L shows higher *a_c_* but is also more compliant than the Blend. Similar to PP-HR, the microlayering process caused changes in the material behaviour of the Blend in the form of increased ductility. Generally, all blends and homogeneous materials lie on a connection line, and a change in material composition (soft phase content) would result in a shift along this line. When comparing ML_512L to this reference line, the low stiffness is clearly outweighed by the increased impact strength, so that the data point lies significantly above the line.

**Figure 6:**
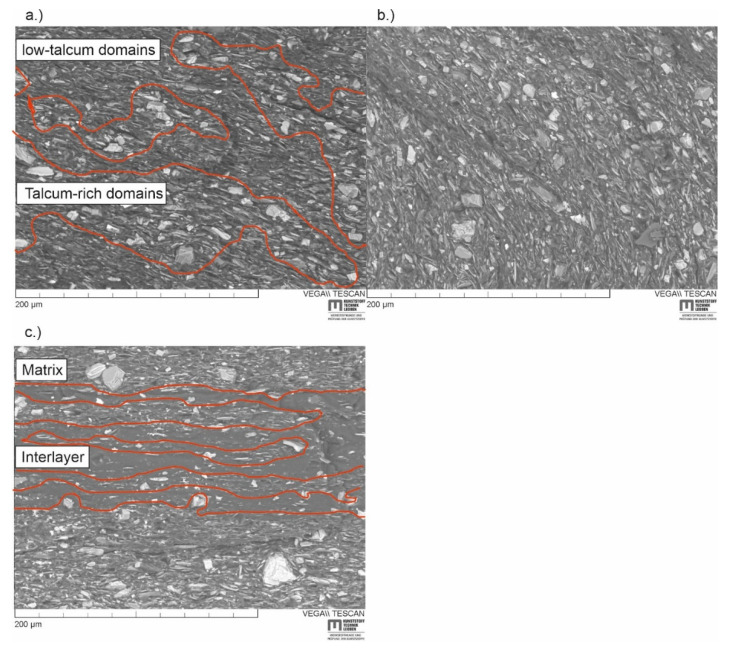
(**a**) SEM-micrographs of a cryofractured Blend and (**b**) Blend_512_L without defined layer structure and (**c**) a cryofractured ML_512L with distinct layers. Talcum-rich domains are separated from low-talcum domains by red lines.

The combination of stiffness and toughness of ML_512L is very different to Blend_512L, highlighting once again the significance of the microstructure in two materials of the same composition. Therefore, the microstructure of the blends and ML materials was analysed using SEM-micrographs. As can be seen in Figure 6a, the Blend shows a microstructure of a randomly distributed matrix phase and soft phase. The domains of matrix material tend to be richer in talcum particles, while domains that are rich in soft phase have little to no reinforcing particles in them (indicated by red lines in Figure 6a). A percolating network of matrix material can form in this material, thus facilitating catastrophic failure. Matrix and IL material in Blend_512L (Figure 6b) are more finely dispersed due to the microlayering process, so that no clear domains can be assigned. This is the reason for the increased strain at break and impact strength, but also the reduced stiffness. While the randomly distributed soft phase has a beneficial effect on toughness, continuous ILs are most effective at stopping cracks. As shown in Figure 6c, bands of IL material act as crack arresters in the multilayer composite ML_512L. The boundaries of matrix material and IL are also indicated by red lines in Figure 6c. For the IL to be effective, the bands of soft material must be continuous throughout the specimen. Otherwise, the maximum possible defect size is not limited to the layer thickness and the benefits to toughness may be lost. This assumption is confirmed by Baer et al. [7], where processing-induced talcum agglomerations interfered with the layer structure, and the benefits to fracture strain were lost.

**Figure 7:**
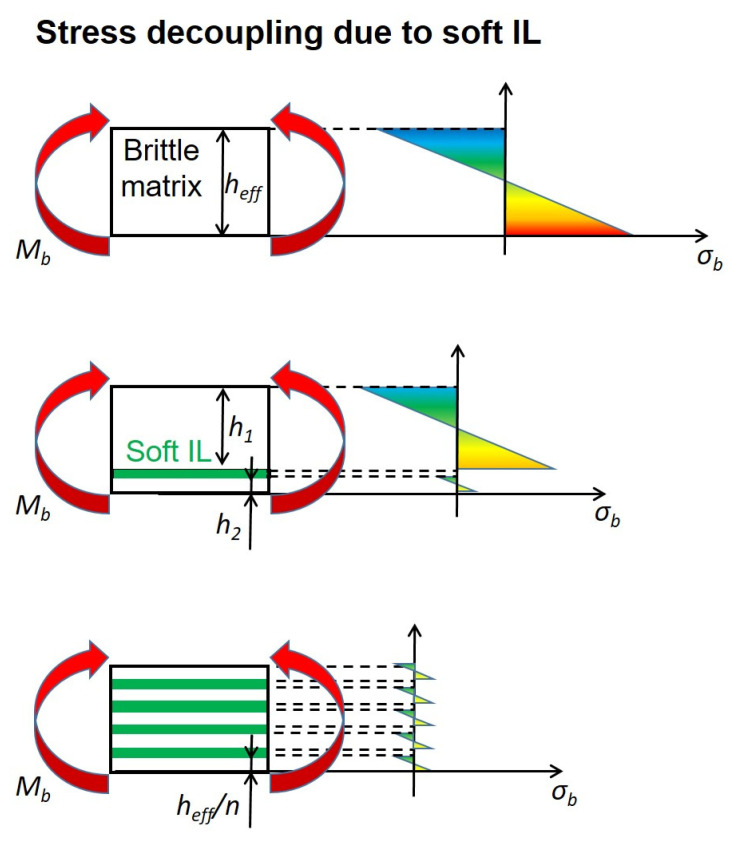
Decoupling of bending stresses due to soft and compliant interlayers in a stiff and brittle matrix. Stress maxima and area moment of inertia decrease as an increasing number of layers are added.

While continuous bands of soft material lead to damage tolerance, they can also alter the stress distribution and failure mechanism of a material. To illustrate this, the matrix ligaments in a multilayer composite are simplified as bending specimens, which is an accurate description, e.g., during a Charpy experiment. If the Young’s Modulus of the IL material is very low compared to that of the matrix, the load bearing matrix ligaments cannot transfer stresses to one another. The stresses of individual ligaments are decoupled, while the deflection remains the same for all layers [38]. Figure 7 depicts such a case, where all individual ligaments behave like separate bending specimens and possess a neutral axis of their own. When adding an increasing number of ILs, the load is distributed more evenly between the various matrix layers and stress maxima are less pronounced. It is more difficult to reach the necessary stresses to break the matrix ligaments. However, a side effect is a strongly reduced bending stiffness. Although a ligament might seem far away from the neutral axis of the entire specimen, no contribution according to the parallel axes theorem (also known as Steiner theorem) is made towards the area moment of inertia. Since the area moment of inertia is strongly dependent on the thickness of the individual layers, *h_eff_*/*n*, a multilayer composite will have less bending stiffness than a comparable bulk material the higher the number of layers, *n*, is. Thus, a material with a microstructure of alternating soft and stiff layers will be tougher but more compliant compared to a blend of similar material composition.

This effect is scarcely described in the literature. To the authors’ knowledge, only increased stiffness has been reported for multilayered materials [7,8], owing to increased particle orientation. In this special case, the benefits of orientation are unfortunately outweighed by the described decoupling effect. Nevertheless, the high mismatch in material properties that causes the stress decoupling is necessary for optimizing fracture toughness. Numerous fracture mechanical publications clearly state that the optimal crack arresting properties of soft layers can only be realized when the IL component is considerably more compliant or has a significantly lower yield stress than the matrix [32,37].

### 3.4. Influence of Loading Direction

The previous sections show that the increase in impact strength of ML composites comes at a high cost in stiffness. This, however, is only true for a bending load, since the main reason behind it is the reduction in the area moment of inertia. Additionally, the pulling through led to a drop-off in force in a Charpy setup (see Figure 3).

**Figure 8:**
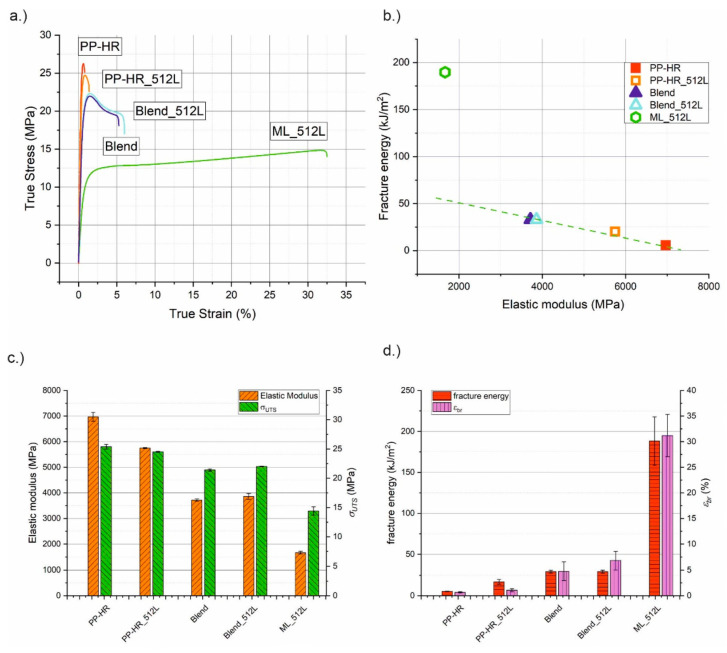
(**a**) Representative true stress–strain curves of all investigated materials in tensile tests and (**b**) an overview of the observed material parameters, where (**c**) the elastic modulus and ultimate tensile strength and (**d**) the fracture energy and strain at break are depicted in more detail.

In a tensile loading situation, we expect similar benefits for fracture toughness and less of a drawback in stiffness. Due to the lack of well-established single edge notch tension standards for polymers, tensile tests were only performed in the unnotched state according to DIN EN ISO 527. True stress–strain curves of all investigated materials as well as a comparison of stiffness and toughness and an overview of material parameters are shown in Figure 8. The exact values as well as the relative values with PP-HR as reference material are presented in Table A2 in Appendix C.

The absolute values of the modulus in the tensile tests were much lower than those evaluated from the Charpy experiments. This is most likely caused by the considerably lower testing speed of the tensile tests. As mentioned earlier, the parameter *E_b_* was primarily used as a ranking parameter and is not recommended for quantitative comparisons to measurements with different experimental conditions. PP-HR shows the highest values for *E* and *σ_UTS_* but also the lowest *ε_b_*_r_ and the lowest fracture energy. These material parameters serve as reference points to compare the other materials. The microlayering process had a beneficial influence on the ductility of PP-HR_512L. Due to an increase in *ε_br_* of 60%, the fracture energy increased by 3.5 times compared to PP-HR. This, however, came at the cost of stiffness, leading to an almost 20% decrease in *E*. For the blends, the introduction of the soft phase reduced *E* to approximately 55% of the reference material, and *σ_UTS_* was also reduced by up to 15%. Owing to a considerable increase in *ε_br_*, the fracture energy increased to almost six times the value of PP-HR. Blend_512L appears to be slightly stiffer, stronger and more ductile than the Blend. However, the differences between the two lies within standards deviations. All in all, the micro layering process showed a negligible influence on the tensile properties of the blends. Incorporating the soft phase in the form of continuous IL brought further benefits to material ductility, namely, *ε_br_* could be increased from less than 1% (PP-HR) to over 30% (ML_512L). The fracture energy surpassed the matrix material by more than 30 times. Despite an identical material composition, the blends were outperformed by a factor of 5, thus reinforcing that the microstructure of the incorporated soft phase is essential.

The reduction in stiffness was not as severe as in the bending setup, so that approximately one quarter of *E* could be retained. In a tension setup, specimen stiffness is only influenced by the total cross-sectional area of the matrix ligaments, which is undiminished by the layered microstructure. Since the more ductile plane stress state is favoured in the thin matrix layers due to the aforementioned stress decoupling, stiffness is still lower than for the blends. The tensile specimens also cannot slide out of the clamping fixture during the experiment. As a result, an actual fracture of the entire specimen is guaranteed, and the full extent of necessary fracture energy can be measured. In addition, a strain hardening could be observed after the yield point in the tensile experiments. Thus, almost 60% of *σ_UTS_* of PP-HR could be preserved. A comparison of fracture energy versus *E* in Figure 8b reveals that ML_512L lies above the trendline of homogeneous materials and blends again. Figure 8c shows, that *E* and *σ_UTS_* could be kept relatively high for ML_512L, while *ε_br_* and the fracture energy excel in comparison to the other materials (see Figure 8d). In conclusion, the toughening effects of the soft IL can be utilized most effectively in a tensile loading situation. While the fracture energy is maximized, the drawbacks for *σ_UTS_* and especially *E* can be kept to a minimum.

## 4. Conclusions

Talcum particle-reinforced polypropylene was investigated through Charpy impact tests and tensile tests. A soft phase of very compliant polypropylene was incorporated in the form of blending as well as continuous soft interlayers (IL).

The materials were assessed regarding stiffness, maximum force and area under the force–displacement curves that were obtained in instrumented Charpy experiments. The influence of notch type (unnotched, Charpy-notch and razor blade notch) and number of layers on impact strength were investigated.

A plateau in impact strength could be reached when the matrix layer thickness was as small or smaller than the size of the largest reinforcing particles, which was 13.6 μm in the investigated talcum particles. The impact strength in the plateau region increased up to 4.5 times compared to specimens with larger matrix layer thicknesses.

Continuous layers led to a high damage tolerance. While multilayered specimens retained over 50% of their original impact strength, blends and homogeneous materials suffered reductions of 80% or more. Thus, introducing the soft phase in the form of continuous layers proved to be a considerably more effective toughening mechanism than the blending of matrix and IL material in a comparable ratio.

The increase in toughness comes at the cost of bending stiffness and maximum force due to a loss in moment of inertia, which is proportional to the number of layers. This is a side effect of stress decoupling between individual matrix layers, since the IL material is too compliant to transfer stresses.

Similar benefits to fracture energy could be found in tensile tests of the multilayer material, but the decrease in strength and stiffness could be partially avoided due to the tensile loading. More specifically, 24% of matrix stiffness could be preserved under tensile loading compared to only 11% in a bending setup.

## Figures and Tables

**Table 1:** Overview of investigated materials including their abbreviation, material composition, description and schematic of microstructure.

Abbreviation	Depiction	Material Composition	Description	Toughening Mechanism
PP-HR	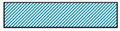	Highly reinforced PP	Homogeneous bulk material	-
PP-HR_512L	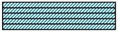	Highly reinforced PP	Bulk material processed in 512 identical layers	High orientation
Blend	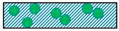	Highly reinforced PP and soft PP (87:13)	Blended material	Soft component
Blend_512L	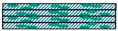	Highly reinforced PP and soft PP (87:13)	Blended material processed in 512 identical layers	Soft component + high orientation
ML_512L	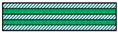	Highly reinforced PP:soft PP (87:13)	Two different materials processed in 512 alternating layers	Soft component + high orientation + defined microstructure

**Figure A1:**
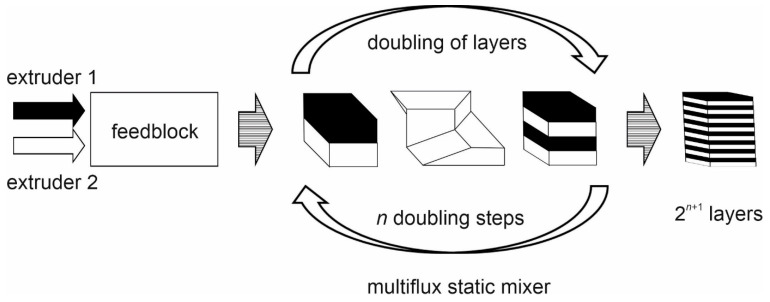
Multilayer extrusion using a multiflux static mixer, adapted from [53].

**Figure A2:**
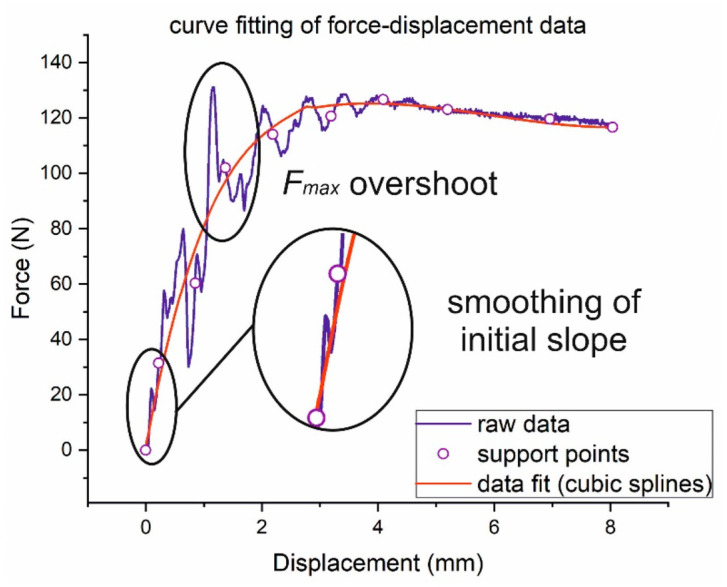
Curve fitting of force–displacement data from instrumented Charpy experiments. Cubic splines were fitted through 10 support points.

**Figure A3:**
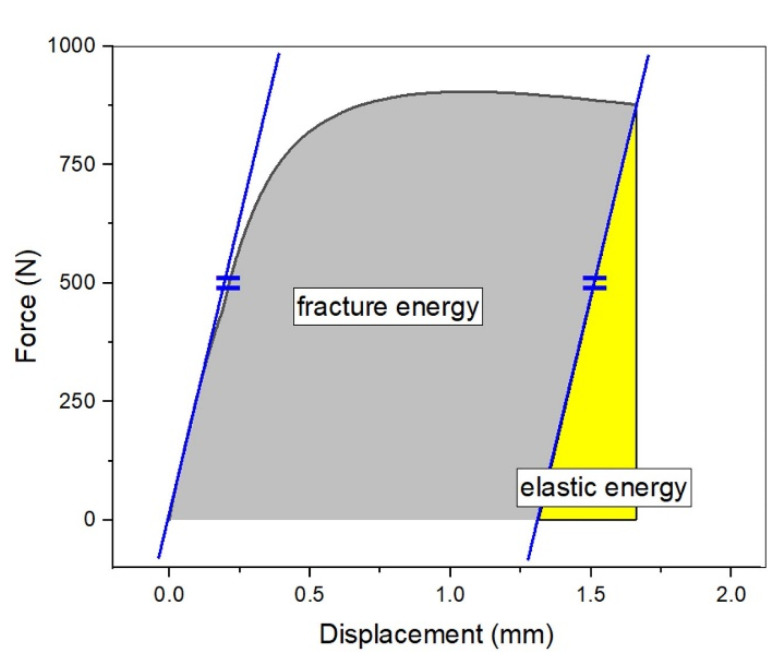
Correction for elastic unloading of the area under the force–displacement curve of tensile tests.

## Data Availability

The data presented in this study are available on request from the corresponding author. The data are not publicly available due to ongoing research.

## Appendix A

The following is a detailed description of the tested materials and the methods used for investigation.

### Appendix A.1. Materials

An overview of all investigated materials is summarized in Table 1. Therein, the material composition, a brief description as well as a schematic drawing of the microstructure are given. All material combinations consist of only two material bases. The matrix material in all investigated combinations consists of a stiff extrusion grade polypropylene, which is highly reinforced with talcum particles (PP-HR, 60 wt% talcum particles). The second material in this study is a compliant type of PP with a very low yield stress, which was used as a toughness enhancing component (termed IL for interlayer) to offer an extreme contrast between the hard and the soft phase. From these two base materials, four more material composites were generated by varying the material composition and processing technique. In a first step, layered composites with different numbers of layers, ranging from 64 to 2048, were prepared. These composites are oriented due to the multilayer extrusion process and exhibit a defined microstructure of alternating layers. All layered materials had the same amount of matrix material and soft interlayer (87% PP-HR to 13% IL). These co-extruded materials were used for a first analysis of the influence of different numbers of layers. Subsequently, a more thorough analysis of the composite with 512 layers was performed. Therefore, this configuration is specifically titled ML_512L. A blend with the same material composition was manufactured to differentiate between the effect of the soft component alone and the effects of orientation and microstructure. This material was simply labelled as Blend. It does not show the high degree of orientation found in a multilayer material, and the microstructure is a random distribution of soft phase within the matrix. The Blend and the homogeneous matrix material were produced also in 512 identical layers (PP-HR_512L, Blend_512L) to observe possible effects of particle orientation due to the microlayering process. All materials were supplied in the form of extruded sheets with a thickness of 4 mm.

### Appendix A.2. Preparation of Materials via Multilayer Co-Extrusion

Multilayer materials were manufactured using two Extrunet ECE-30 extruders and a multiflux static mixer with a modular design. Melt strands of the matrix and IL material are joined in a feedblock after passing the extruders. After passing the feedblock, one module of the static mixer divides the two layers of melt vertically, before placing the halves on top of each other horizontally (see Figure A1). Thus, each additional module doubles the number of layers, so the final number of layers is 2^*n*+1^, with *n* being the number of modules. This processing routine has proven to provide neatly stacked polypropylene multilayer materials with uninterrupted continuous layers [12,13].

### Appendix A.3. Specimen Preparation

As mentioned above, materials were supplied as 4 mm thick plates. From these, Charpy specimens according to DIN EN ISO 179-1 were manufactured with dimensions of 80 × 10 × 4 mm^3^. For every material, unnotched, Charpy-notched and razor blade notched specimens were prepared. As shown in Figure 1, all notches were introduced flatwise, so the effect of a layered plate could be observed. A saw blade with a crack tip radius of 0.25 mm was used to introduce the Charpy-notches. A broaching tool was used to introduce the razor blade notches [55,56,57]. All notches were prepared to have an overall depth of 0.3 mm, and all specimens were manufactured parallel to extrusion direction. Additionally, tensile test specimens (Type 1A) were prepared in order to be tested according to DIN EN ISO 527.

### Appendix A.4. Charpy Measurements

All experiments were performed using a Zwick/Roell HIT25P pendulum. Tests were performed according to DIN EN ISO 179-1fA for notched and DIN EN ISO 179-1fU for unnotched specimens. For each material, 5 individual specimens were tested using a 2J pendulum. In all cases, a flatwise specimen orientation was used, so failure had to progress through all individual layers.

### Appendix A.5. Instrumented Charpy Experiments

All experiments were performed using a Zwick/Roell HIT25P with an instrumented 2J pendulum. The pendulum was equipped with a piezo-electric load cell within the fin, and measurements were taken with a sampling rate of 2 MHz. For each measurement series, a total of six specimens were measured with the exception of the multilayered composites. In these cases, only two specimens could be measured due to limited material supply. The specimen orientation was also flatwise in all cases.

### Appendix A.6. Data Treatment

Due to high oscillation during the instrumented impact tests, the maximum force, *F_max_*, and initial slope could not be reliably determined from the raw data (see Figure A2). Therefore, a total of 10 support points was set manually for each curve. The support points were then used to create a fit of cubic splines, which was later used to determine the maximum force and initial slope.

### Appendix A.7. Normalization of Parameters

From the specimen width, *b*, and the effective thickness, *h_eff_*, an effective cross-sectional area *A_eff_* was calculated for all specimens (see Equation (A1)). For unnotched specimens, *h_eff_* is equal to the total plate thickness *h*. For notched specimens, the remaining ligament length at the root of the notch was taken as *h_eff_*. The area *U* was obtained through integration of the force–displacement curve. For this integration, the raw data and not the fits were used. Corrections for elastic unloading were forgone, since the initial slope could not be reliably determined for all specimens. This was mostly the case for the more brittle matrix materials under the influence of a notch, where standard deviations were large even after the curve fitting. The impact strength, *a_c_*, was evaluated for unnotched, Charpy-notched and razor blade notched specimens (Equation (A2)). The maximum force and initial slope were determined from the fitted curves. The slope was calculated from the differences in force, Δ*F*, and displacement, Δ*v*, in the initial, linear part of the curve. To be exact, the slope was calculated between 0.085 and 0.427 mm deflection, which translates to 0.05% and 0.25% maximum strain. From the initial slope in the unnotched state the bending modulus, *E_b_* was calculated according to Equation (A3) to compare the stiffness of the materials. The calculation is in agreement with DIN EN ISO 178, although the testing speed in this case is much higher than in conventional three-point-bending tests. *E_b_* should, therefore, be seen as a ranking parameter in these specific considerations and cannot be quantitatively compared to values obtained from quasi-static experiments. In order to remove the influence of specimen geometry, the maximum force, *F_max_*, was divided by *A_eff_* of each individual specimen. The formula for the normalised maximum Force, $F_{max}^{norm}$, is shown in Equation (A4).

(A1) $$A_{eff}=\;bh_{eff}$$

(A2) $$a_{c}=\;\frac{U}{A_{eff}}$$

(A3) $$E_{b}\;=\;\frac{\Delta F}{\Delta v}\frac{L^{3}}{4bh\begin{array}{c} 3\\ eff \end{array}}$$

(A4) $$F\begin{array}{c} norm\\ max \end{array}\;=\;\frac{F_{max}}{A_{eff}}$$

### Appendix A.8. Tensile Tests

All tensile tests were performed according to DIN EN ISO 527 using a Zwick Z250 electrodynamic tensile testing machine, equipped with a 10 kN loadcell. In addition to the Young’s modulus, *E*, the ultimate tensile strength, *σ_UTS_*, and the elongation at break, *ε_br_*, were measured. In the considerations regarding tensile properties, the area under the force–displacement curve was corrected for elastic unloading (see Figure A3) and taken as fracture energy, which acted as measure for toughness.

### Appendix A.9. SEM-Micrographs

To investigate the microstructure of the samples, scanning electron microscopy was applied. All samples were sputtered with a thin layer of gold to achieve surface conductivity. SEM-graphs were then created using a TESCAN Vega II, whereas the fractographs were generated from the backscatter electron to give an improved contrast between talcum particles and polymer matrix.

## Appendix B

**Table A1:** Results obtained from instrumented Charpy experiments including *E_b_*, $F_{max}^{norm}$ and *a_c_*.

Material	*E_b_* (MPa)	Notch Type	$F_{max}^{norm}$ (N/mm^2^)	*a_c_* (kJ/m^2^)
PP-HR	17,475 ± 4056	NoNotch	3.07 ± 0.66	2.66 ± 0.40
		Charpy	2.36 ± 0.48	0.46 ± 0.30
		Razor	1.42 ± 0.68	0.25 ± 0.14
PP-HR_512L	14,141 ± 794	NoNotch	3.08 ± 0.31	3.86 ± 0.35
		Charpy	2.39 ± 0.15	0.78 ± 0.11
		Razor	2.33 ± 0.16	0.82 ± 0.06
Blend	9635 ± 894	NoNotch	2.64 ± 0.06	7.14 ± 0.53
		Charpy	2.59 ± 0.24	2.21 ± 0.45
		Razor	2.33 ± 0.34	1.52 ± 0.12
Blend_512L	9258 ± 1268	NoNotch	2.67 ± 0.13	9.39 ± 1.63
		Charpy	2.35 ± 0.26	2.51 ± 0.34
		Razor	2.55 ± 0.19	1.58 ± 0.13
ML_512L	1903 ± 457	NoNotch	0.88 ± 0.04	30.99 ± 0.80
		Charpy	0.71 ± 0.05	16.95 ± 0.26
		Razor	0.79 ± 0.02	15.53 ± 0.62

## Appendix C

**Table A2:** Material parameters obtained from tensile tests.

	*E* (MPa)	*ε_br_* (MPa)	*σ_UTS_* (MPa)	Fracture Energy Per Area (kJ/m^2^)
	Absolute Values:			
PP-HR	6967 ± 173	0.67 ± 0.11	25.4 ± 0.4	5.3 ± 0.3
PP-HR_512L	5748 ± 23	1.08 ± 0.30	24.5 ± 0.1	16.9 ± 2.9
Blend	3714 ± 45	4.75 ± 1.79	21.4 ± 0.2	29.4 ± 1.6
Blend_512L	3862 ± 111	6.81 ± 1.76	22.0 ± 0.04	29.4 ± 1.7
ML_512L	1666 ± 50	31.19 ± 4.13	14.4 ± 0.7	188.2 ± 29.6
	Relative values (PP-HR as reference):			
PP-HR	1	1	1	1
PP-HR_512L	0.83	1.61	0.96	3.54
Blend	0.53	7.09	0.84	5.79
Blend_512L	0.55	10.16	0.87	5.79
ML_512L	0.24	46.55	0.57	33.28
